# A Comparative Analysis of Resistance Patterns in Rifampin-Resistant Tuberculosis: Mutations Within and Outside the Rifampicin Resistance-Determining Region

**DOI:** 10.7759/cureus.93975

**Published:** 2025-10-06

**Authors:** Haris Saeed, Muhammad Adnan, Tayyeba Komal, Sana Rehman, Muhammad Kashif Munir

**Affiliations:** 1 General Internal Medicine, Combined Military Hospital (CMH) Lahore Medical College and Institute of Dentistry, Lahore, PAK; 2 Health Research Institute, National Institute of Health, Islamabad, PAK; 3 Pathology, Services Institute of Medical Sciences, Lahore, PAK; 4 Health Research Institute-National Institute of Health Tuberculosis Research Center, King Edward Medical University, Lahore, PAK

**Keywords:** genexpert mtb/rif assay, rifampin isoniazid, rifampin resistant, rpob gene, tuberculosis

## Abstract

Background

Rifampicin resistance in tuberculosis (TB), often linked with isoniazid resistance, defines multidrug-resistant TB. The rifampicin resistance-determining region (RRDR) of the *rpoB* gene is a recognized hotspot, yet mutations outside the RRDR and their clinical relevance remain underexplored. This study aimed to compare drug resistance patterns associated with *rpoB* gene mutations occurring within and outside RRDR in patients with rifampicin-resistant pulmonary tuberculosis (RR-TB).

Methodology

This cross-sectional analytical study included 170 GeneXpert-confirmed RR-TB cases from Mayo Hospital/ King Edward Medical University, Lahore, from August 2020 to July 2022. Probe-missing patterns were recorded, and phenotypic drug susceptibility testing using the proportion method on Lowenstein-Jensen medium was performed. Chi-square test and logistic regression analysis were performed to compare resistance patterns between groups, with p-values ≤0.05 considered significant.

Results

Among 170 cases, the overall mean age was 35.45 ± 16.11 years, 90 (52.9%) were males, 156 (91.8%) had mutations within the RRDR, and 14 (8.2%) had mutations outside the RRDR. Age, sex, or body mass index demonstrated no association with non-RRDR mutations (all p > 0.05). Isoniazid resistance was higher in RRDR cases (84.0% vs. 57.1%; p = 0.025). Amikacin resistance was only observed in non-RRDR cases (0.0% vs. 7.1%; p = 0.024). Out of 133 MDR-TB cases, 125 (94.0%) had missing probes A to E, including 109 (82.0%) with missing probe E. However, 8 (6.0%) MDR-TB cases had no missing probes.

Conclusions

Mutations in the RRDR remained the primary drivers of MDR-TB. However, mutations outside the RRDR suggested a potential association with amikacin resistance, necessitating expanded molecular surveillance and larger studies for validation.

## Introduction

Tuberculosis (TB) remains one of the leading infectious causes of death worldwide, surpassing both malaria and human immunodeficiency virus infection in annual mortality. According to the World Health Organization’s Global Tuberculosis Report 2024, approximately 10.8 million people developed TB in 2023, and around 1.25 million people died from the disease. Globally, only about 44% of patients with multidrug-resistant (MDR) or rifampicin-resistant (RR) TB were diagnosed and treated. Pakistan is one of the five countries that together account for more than half of the global TB burden. Each year, Pakistan records an estimated 420,000 new TB cases, making the disease one of its most serious public health concerns [1]. These statistics reveal a medical and humanitarian crisis, underscoring the urgent need for improved prevention, early diagnosis, and effective treatment [2]. The rapid molecular detection of RR-TB using the Xpert MTB/RIF assay has revolutionized diagnostics by delivering reliable results within hours [3]. The development of new tools, such as simple finger-stick blood tests based on gene expression and rapid antigen detection kits, has made diagnosis faster and more accessible [4]. Such diagnostic modalities are important, particularly for Pakistan, where early detection can help stop the spread of TB, improving patient care while reinforcing the country’s efforts to overcome a long-standing epidemic.

The GeneXpert® MTB/RIF assay simultaneously detects *Mycobacterium tuberculosis* and rifampicin resistance by targeting DNA mutations within the 81-bp rifampicin resistance-determining region (RRDR) of the *rpoB* gene, shown in the Appendices (Figure) [5], where over 95% of resistance mutations occur. The introduction of GeneXpert® has transformed the diagnosis of TB and drug-resistant TB by addressing the limitations of conventional smear and culture methods [2]. This assay targets DNA sequences associated with rifampicin resistance, with more than 95% of resistant mutations occurring within the 81-bp RRDR of the *rpoB* gene. Using a single polymerase chain reaction (PCR) amplicon, the system simultaneously detects *Mycobacterium tuberculosis* and rifampicin resistance. It utilizes five distinctively labeled hybridization probes (A-E), each designed to bind to a specific sequence within the RRDR. A loss of signal from any probe indicates resistance to rifampicin [6]. Besides identifying rifampicin resistance within the RRDR, the assay can also detect resistance outside this region [7]. Although regional variations in probe missing patterns have been reported [8], the differences in phenotypic resistance to rifampicin and other first-line anti-TB drugs between mutations within and outside the RRDR in Pakistan remain inadequately understood. Rifampicin and isoniazid are the two most potent first-line anti-TB agents. Resistance to both, irrespective of additional resistance to other first-line drugs, is classified as MDR-TB [9]. The indication between rifampicin and isoniazid is already well established and effectively applied in the management of drug-resistant and MDR-TB cases [9, 10]. Similarly, a question arises about the potential indications within the existing *rpoB* gene model, along with the possibility that rifampicin resistance may also serve as an indication of resistance to other anti-TB drugs, worth further investigation. Moreover, rifampicin-related indications may also help guide the use of other first-line and second-line anti-TB drugs. A more in-depth consideration of missing probe patterns could contribute to establishing more precise objectives, particularly in refining diagnostic strategies and guiding targeted approaches for the management of drug-resistant TB. Therefore, this study compared drug resistance patterns associated with *rpoB* gene mutations occurring within and outside the RRDR in patients with RR-TB.

## Materials and methods

This cross-sectional analytical study included 170 GeneXpert-confirmed RR-TB cases from the Pulmonology Outpatient Department (OPD), Mayo Hospital/ King Edward Medical University (KEMU), Lahore, from August 2020 to July 2022. This public facility operates under the Provincial TB Control Program (PTP) of Punjab, which provides free diagnostic and treatment services. The patient population attending this facility is primarily drawn from low socioeconomic groups, often characterized by low literacy, employment as daily laborers, and a high occurrence of comorbid diseases. Ethical approval (approval number: 438/RC/KEMU dated July 4, 2020) was sought from the Institutional Review Board of KEMU Lahore. Informed consent was obtained from all volunteer participants.

Of the 6,000 suspected patients who visited the facility during the study period, 1,080 (18.0%) were diagnosed as MTB positive. Of them, 170 (15.7%) RR-TB cases were purposively included in the study. RR-TB patients aged >18 years and of any sex were consecutively enrolled in the study. Exclusion criteria were patients taking anti-tubercular treatment to treat MDR-TB for more than two months, terminal illnesses, or culture-negative cases. The minimum sample size was estimated using a 95% confidence level, 5% absolute precision, and 12.5% expected RR-TB without missing probe outside the RRDR [7].

A specially designed questionnaire was used to collect age (years), sex, marital status, education, body mass index (BMI, kg/m^2^), acid-fast bacilli (AFB) microscopy, and GeneXpert results. The GeneXpert MTB/RIF assay (Cepheid, USA) was used to detect MTB and RIF resistance. Sputum samples were decontaminated and liquefied using a modified Petroff method. The treated sample mixtures were transferred directly into GeneXpert cartridges and loaded into a fully automated GeneXpert instrument for DNA extraction, purification, PCR amplification, and MTB/RIF detection. The assay utilizes five molecular beacon probes (A, B, C, D, and E) that cover the 81-bp RRDR of the *rpoB* gene. If all five probes bind correctly and fluoresce, it indicates a mutation outside the RRDR. If one or more probes fail to bind/fluoresce, it indicates a mutation in the RRDR.

Smear grading of slides stained with Ziehl-Neelsen (ZN) was also recorded to observe bacillary load. A first morning guided sputum sample was collected from each patient for culture and drug susceptibility testing (DST). The proportion method was used for conventional culture and phenotypic DST on Lowenstein-Jensen (LJ) medium. The DST included first-line anti-TB drugs such as Isoniazid (0.2 μg/mL), rifampicin (40.0 μg/mL), ethambutol (2.0 μg/mL), and streptomycin (4.0 μg/mL); second-line drugs included amikacin (4.0 μg/mL), kanamycin (30.0 μg/mL), capreomycin (40.0 μg/mL), ofloxacin (2.0 μg/mL), and ethionamide (40.0 μg/mL). The growth of *Mycobacterium tuberculosis* in drug-containing media was compared with drug-free controls to classify isolates as resistant or susceptible. Drug resistance was defined according to phenotypic DST results on LJ media. MDR-TB was defined as resistance to at least both isoniazid and rifampicin [11].

Data were entered and analyzed using SPSS software version 25 (IBM Corp., Armonk, NY, USA). Descriptive results are presented in frequency and percentage for categorical variables and mean ± standard deviation for continuous variables. Chi-square test and logistic regression analysis performed to compare resistance patterns between groups, with p-values ≤0.05 considered significant.

## Results

The study included 170 RR-TB cases, of whom 90 (52.9%) were males, 58 (34.1%) were unmarried, and 60 (35.3%) were illiterate. The mean age was 35.45 ± 16.11 years, and the BMI was 18.52 ± 3.66 kg/m². The proportion of the age group >32 years was 84 (49.4%), and 101 (59.4%) cases were underweight. AFB smear grading 2+ was the most common result in 68 (40.0%) cases. GeneXpert low positivity (82, 48.2%) was the most frequent, followed by very low positivity (37, 21.8%), as shown in Table 1.

**Table 1:** Demographic and clinical characteristics of RR-TB patients recorded upon enrollment in the study. BMI: body mass index; RR-TB: rifampicin-resistant tuberculosis

Variables	Categories	Total (n = 170)			
		N	%	Mean	SD
Sex	Male	90	52.9	-	-
	Female	80	47.1		
Age (years)	≤32	86	50.6	35.45	16.11
	>32	84	49.4		
Marital status	Married	112	65.9	-	-
	Unmarried	58	34.1		
Education	Illiterate	60	35.3	-	-
	Primary	37	21.8		
	Middle	27	15.9		
	High	40	23.5		
	Higher above	6	3.5		
BMI (kg/m^2^)	Underweight	101	59.4	18.52	3.66
	Normal	58	34.1		
	Overweight	9	5.3		
	Obese	2	1.2		
Smear result	Scanty	9	5.3	-	-
	1+	59	34.7		
	2+	68	40.0		
	3+	34	20.0		
GeneXpert result	Very low	37	21.8	-	-
	Low	82	48.2		
	Medium	30	17.6		
	High	21	12.4		

The frequency of RRDR was 156 (91.8%) and of non-RRDR was 14 (8.2%). There was no statistically significant association between demographics and risk of non-RRDR mutations, as shown in Table 2.

**Table 2:** Comparison of demographic and clinical characteristics between RRDR and non-RRDR groups and risk factors of mutations outside the RRDR in RR-TB patients. RRDR: within the rifampicin resistance-determining region; non-RRDR: outside the rifampicin resistance-determining region; BMI: body mass index; RR-TB: rifampicin-resistant tuberculosis

Variables	Categories	RRDR (n = 156)		Non-RRDR (n = 14)		Adjusted odds	P-value
		N	%	N	%		
Age (years)	≤32	76	88.4	10	11.6	1	0.100
	>32	80	95.2	4	4.8	0.254 (0.050, 1.302)	
Sex	Male	82	91.1	8	8.9	1	0.442
	Female	74	92.5	6	7.5	0.599 (0.162, 2.213)	
Marital status	Married	103	92.0	9	8.0	1	0.369
	Unmarried	53	91.4	5	8.6	0.497 (0.108, 2.287)	
Education	Literate	98	89.1	12	10.9	1	0.246
	Illiterate	58	96.7	2	3.3	0.375 (0.072, 1.963)	
BMI (kg/m^2^)	Normal weight	51	87.9	7	12.1	1	0.129
	Underweight	95	94.1	6	5.9	0.384 (0.112, 1.319)	
	Overweight and obese	10	90.9	1	9.1	0.710 (0.073, 6.911)	0.768

Comparison of phenotypic DST patterns between RRDR and non-RRDR mutations showed that isoniazid resistance was significantly higher in RRDR cases (131, 84.0%) compared to non-RRDR (8, 57.1%) (p = 0.025). No significant difference was observed for rifampicin, ethambutol, streptomycin, kanamycin, ofloxacin, or ethionamide resistance between the two groups. However, a notable finding was noted for amikacin, where all RRDR cases were sensitive (156, 100%), while 1 (7.1%) non-RRDR case was resistant, yielding a statistically significant difference (p = 0.024). Resistance to other second-line drugs, including capreomycin, remained rare and showed no meaningful difference across mutation groups, as presented in Table 3.

**Table 3:** Comparison of drug resistance patterns against first and second-line anti-TB drugs between RRDR and non-RRDR groups. RRDR: within the rifampicin resistance-determining region; non-RRDR: outside the rifampicin resistance-determining region; TB: tuberculosis

Anti-TB drugs		RRDR		Non-RRDR		Chi-square value	p-value
		n	%	n	%		
Isoniazid	Sensitive	25	16.0	6	42.9	5.049	0.025
	Resistant	131	84.0	8	57.1		
Rifampicin	Sensitive	10	6.4	1	7.1	0.011	0.916
	Resistant	146	93.6	13	92.9		
Ethambutol	Sensitive	130	83.3	12	85.7	0.055	0.815
	Resistant	26	16.7	2	14.3		
Streptomycin	Sensitive	111	71.2	12	85.7	1.537	0.215
	Resistant	45	28.8	2	14.3		
Kanamycin	Sensitive	155	99.4	14	100.0	0.172	0.678
	Resistant	1	0.6	0	0.0		
Ofloxacin	Sensitive	122	78.2	9	64.3	1.281	0.258
	Resistant	34	21.8	5	35.7		
Ethionamide	Sensitive	144	92.3	13	92.90	0.006	0.940
	Resistant	12	7.7	1	7.1		
Amikacin	Sensitive	156	100.0	13	92.9	5.061	0.024
	Resistant	0	0.0	1	7.1		
Capreomycin	Sensitive	155	99.4	14	100.0	0.172	0.678
	Resistant	1	0.6	0	0.0		

Overall, 5 (2.9%) cases showed no phenotypic drug resistance. MDR-TB was noted in 133 (78.2%) cases. On the other hand, the missing probe E (133, 78.2%) was revealed as the hotspot region for drug-resistant TB. Out of 133 MDR-TB cases, 125 (94.0%) had missing probes A to E, including 109 (82.0%) with missing probe E. However, 8 (6.0%) MDR-TB cases had no missing probe, as illustrated in Table 4.

**Table 4:** Comparison of missing probes patterns between no-, mono-, and multi-drug resistant groups NR: no resistance; Mono: isoniazid or rifampicin resistance; MDR: multi-drug resistance; DST: drug susceptibility testing

Missing probe	n = 170	Phenotypic DST					
		NR 5 (2.9%)		Mono 32 (18.8%)		MDR 133 (78.2%)	
Probe A	9 (5.3%)	0	0.0%	3	9.4%	6	4.5%
Probe B	5 (2.9%)	1	20.0%	0	0.0%	4	3.0%
Probe C	6 (3.5%)	0	0.0%	1	3.1%	5	3.8%
Probe D	3 (1.8%)	0	0.0%	2	6.3%	1	0.8%
Probe E	133 (78.2%)	3	60.0%	21	65.6%	109	82.0%
No probe missing	14 (8.2%)	1	20.0%	5	15.6%	8	6.0%

## Discussion

Multiple TB strains with different virulence and resistance patterns affect their spread across regions [12]. Both host-related and environmental factors are considered key contributors to the transmission of diverse MTB strains [13]. A crucial part of any TB control program is identifying the mode and site of transmission. This helps prevent further spread and progression to active TB by detecting new infections, ensuring timely treatment, and monitoring treatment completion, leading to a cure. These strategies also help trace transmission links and reveal cases where related patients were infected by different strains [14]. The evaluation of DST is a vital component in monitoring treatment completion and achieving cure of TB. This has remained a critical challenge in maintaining a standardized treatment regimen. In the case of TB and drug-resistant TB, multiple drug combinations are often required to effectively manage the disease, largely due to the slow growth of MTB and the need for medications that act at different stages of its growth under varying conditions [15]. Although the RRDR is recognized as a hotspot region for rifampicin resistance, the missing probe E has emerged as the primary hotspot within the RRDR, accounting for 78.2% in this study. These findings are consistent with results reported both within this region and in other parts of the world [16-18].

Similarly, the missing probe E was strongly associated with the classification of isolates as true MDR, conferring resistance in 82.0% of all MDR isolates analyzed in this study. In a study from Azad Jammu & Kashmir, Pakistan, the missing probe E was the most frequent (34%), followed by probe D (26%), probe B (15.4%), probe A (9.4%), and probe C (3%) [19]. In contrast, the present study found twice higher rate of the missing probe E (78.2%) and substantially lower rates for other missing probes (A-D).

In this study, 78.2% RR cases detected by GeneXpert also showed concurrent isoniazid resistance on the drug proportion method, confirming MDR-TB. An overall rate of 83.3% was observed for rifampicin resistance, also resistant to isoniazid, while six (3.5%) cases reported as rifampicin-resistant on GeneXpert were sensitive on culture. Similarly, isoniazid resistance remained significantly high (p = 0.025) in the RRDR group compared to the non-RRDR group. These findings align with a previous report [20], showing 81.25% in agreement, though that study noted a higher proportion (11.9%) of RR cases by GeneXpert that were susceptible on culture. In contrast, another study reported a higher coexistence rate of 90% [21]. In the present results, GeneXpert® and the standard proportion method showed 93.5% conformity for rifampicin resistance, with 6.5% being sensitive. Culture-based phenotypic DST remains the gold standard, as it covers a broader range of drugs and concentrations, while rifampicin DST is considered reliable but not fully optimized [22]. A previous study revealed agreement rates by drug proportion methods as 78.7% for the disputed group (conferring 511Pro, 516Tyr, 526Asn, 526Leu, 533Pro, and 572Phe mutations in the *rpoB* Gene) remained significantly low at 96.7% in the group of other mutations [23].

Resistance to rifampicin is a multifaceted topic that extends beyond the clinical mechanisms, most often arising from genetic alterations in the β-subunit of bacterial RNA polymerase. Research on resistant polymerases has also provided valuable insights into the enzyme’s structural organization, the complexities of the transcription process, and its involvement in diverse physiological pathways [24]. The GeneXpert® assay is widely utilized as a rapid diagnostic tool for predicting MDR-TB, primarily through the detection of rifampicin resistance [10,25]. However, its potential for predicting resistance to other first-line and second-line anti-TB drugs has not been comprehensively explored. Advancing this area requires not only extensive laboratory investigations but also robust and well-focused data analyses to identify reliable predictive markers and enhance the assay’s clinical utility. A noteworthy and unique finding of this study is the significant association of amikacin resistance (p = 0.024) in the non-RRDR group, suggesting that rifampicin-resistant cases outside the RRDR may also exhibit resistance to amikacin. This finding indicates a potential marker for cross-resistance, underscoring the need for further research to explore its diagnostic and therapeutic implications. However, the study is limited by the relatively small size of the non-RRDR group, which may affect the strength and reliability of comparisons across different levels. Therefore, larger, multi-centered studies are necessary to validate and expand upon this preliminary evidence.

The strengths of this study include its significant focus on understudied non-RRDR mutations, the adoption of phenotypic DST as the gold standard comparison, and the inclusion of MDR profiling. Conversely, the limitations involve the small non-RRDR group, which means the study is underpowered for robust statistical comparisons; a selection bias due to single-center, hospital-based sampling that limits generalizability; and critically, the absence of genomic sequencing to confirm mutation sites or strain lineages.

## Conclusions

The RRDR remains the most critical region associated with rifampicin resistance also serves as a strong predictor of concurrent isoniazid resistance, thereby confirming the classical definition of MDR-TB. However, resistance occurring outside the RRDR region presents a distinct pattern, as suggested by a potential association with amikacin resistance. This initial finding suggests that while RRDR mutations largely determine rifampicin-isoniazid cross-resistance, non-RRDR mutations may potentially serve as markers for broader drug resistance, particularly to second-line agents such as amikacin. Such insights emphasize the need for more extensive molecular and phenotypic studies to better characterize these resistance patterns and improve treatment strategies for drug-resistant TB.

## Figures and Tables

**Figure 1:**
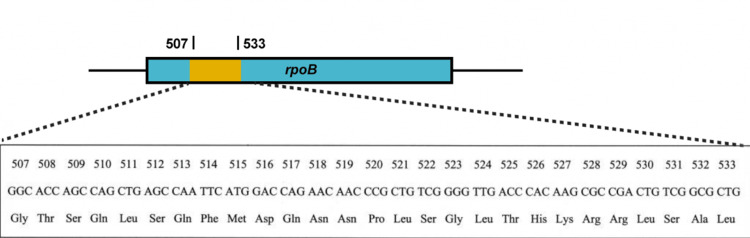
The rpoB gene showing the rifampicin resistance-determining region. Adopted from the National TB Elimination Program (NTEP) [5].

## Appendices

Proforma

Comparative analysis of resistance patterns in rifampin-resistant tuberculosis: Mutations within and outside the rifampicin resistance-determining region

Full Name: ______________________________________________ ID __________________

Contact/Address: _____________________________________________________________

1. Age: _____________ years

2. Sex: 0. Male 1. Female

3. Marital status: 0. Married 1. Unmarried

4. Education: 0. Illiterate 1. Primary 2. Middle 3. High 4. Higher above

5. BMI: _____________ kg/m2

6. Smear: 0. Scanty 1. 1+ 2. 2+ 3. 3+

7. Xpert: 0. Very low 1. Low 2. Medium 3. High

8. Missing probe: 0. None 1. A 2. B 3. C 4. D 5. E

9. DST:

a) Isoniazid: 0. Sensitive 1. Resistant

b) Rifampicin: 0. Sensitive 1. Resistant

c) Ethambutol: 0. Sensitive 1. Resistant

d) Streptomycin: 0. Sensitive 1. Resistant

e) Kanamycin: 0. Sensitive 1. Resistant

f) Ofloxacin: 0. Sensitive 1. Resistant

g) Ethionamide: 0. Sensitive 1. Resistant

h) Amikacin: 0. Sensitive 1. Resistant

i) Capreomycin: 0. Sensitive 1. Resistant
